# Combined Transradial and Upper Extremity Transvenous Accesses in the Treatment of Carotid-Cavernous Fistulae: Two Case Reports and a Literature Review

**DOI:** 10.7759/cureus.24094

**Published:** 2022-04-13

**Authors:** Denis Babici, Phillip M Johansen, Octavio Carranza, Brian Snelling

**Affiliations:** 1 Neurology, Florida Atlantic University Charles E. Schmidt College of Medicine, Boca Raton, USA; 2 Neurosurgery, Boca Raton Regional Hospital, Boca Raton, USA

**Keywords:** venogram, arteriogram, carotid cavernous fistula, transradial access, transvenous access

## Abstract

The expansion of indications for neurointerventional procedures, combined with the need to treat a diverse patient population, has driven a need for broader access options. Concurrent arterial and venous access is often necessary for the diagnosis and treatment of various neurovascular diseases. Although complication rates are low, life-threatening severe complications have been reported with these access methods. Moreover, venous access through traditional routes can be challenging in patients with large body habitus. There is a growing trend of utilizing radial artery access for neuroendovascular procedures due to the increased ease of access and similar efficacy. Nevertheless, the use of upper limb veins in neurointerventional procedures is still rare. Upper extremity transvenous access (UETV) has recently emerged as an alternative strategy for neurointerventionalists, but data are limited. This study reports two cases of successful combined upper extremity transvenous access (TVA) and transradial access (TRA) in the treatment of carotid artery-cavernous fistulae. Combined TRA and UETV is a feasible, promising access strategy for patients and may also confer the same safety and patient satisfaction outcomes that have been seen with TRA. Further studies are needed to elucidate the exact impact this strategy has on patient outcomes and satisfaction.

## Introduction

Radial artery access for transarterial procedures has gained recent traction among neurointerventionalists due to technical feasibility, decreased patient morbidity, and improved patient satisfaction. Upper extremity transvenous access (UETV) has also emerged as an alternative strategy for neurointerventionalists, but data are limited [1]. The authors’ objectives were to quantify the use of UETV and measure failure and access complications. While many neurointerventional procedures require arterial access, transvenous access (TVA) has diagnostic and therapeutic utilization, ranging from diagnostic venography and venous sampling to embolization, thrombectomy, and stenting. TVA is considered the route of choice for the treatment of a wide range of neuroendovascular conditions, including inferior petrosal sinus sampling, mechanical thrombectomy in cerebral venous sinus thrombosis, venous sinus stenting for idiopathic intracranial hypertension, embolization of vein of Galen malformations, and many dural arteriovenous and carotid-cavernous fistulae (CCF) [2-4]. Upper extremity transvenous access (UETV) has been shown in recent studies to be a promising method of access that may confer the same safety, cost, and benefits as its arterial counterpart, transradial access (TRA). While there has been a dramatic increase in TRA in neurointerventional publications in the past few years, studies regarding UETV or combined TRA and UETV are lacking. The authors present the cases of two patients with CCF who were treated successfully with combined TRA and UETV.

## Case presentation

Case 1

**Figure 1:**
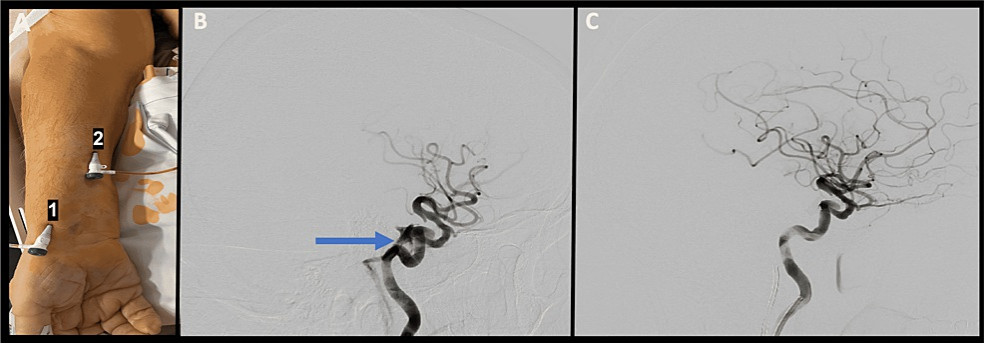
Lateral angiogram A: Transradial access (1) and transvenous access (2) through the cephalic vein. B: Lateral angiogram depicting direct carotid-cavernous fistula arising from the posterior genu of the cavernous internal carotid artery and draining into the inferior petrosal sinus (blue arrow). C: Lateral angiogram after coil embolization showing obliteration of the carotid-cavernous fistula.

An 82-year-old male underwent successful mechanical thrombectomy for large vessel occlusion of the right middle cerebral artery (MCA) M1 segment. During the procedure, he sustained perforation of the cavernous internal carotid artery (ICA) at the posterior genu due to severe tortuosity and underlying atherosclerosis, resulting in a direct CCF (Figure 1B). The next day, the patient was noted to have worsened visual acuity of the right eye along with chemosis and proptosis. The senior author was consulted, and the decision was made to perform embolization of the CCF using combined TRA and UETV (Figure 1A), with hopes of preventing further loss of vision and restoring baseline visual acuity. Transvenous coil embolization to obliterate the CCF went without complications (Figure 1C), and the patient’s vision was noted to have returned to his preoperative baseline the following day. No access site complications were encountered.

Case 2

**Figure 2:**
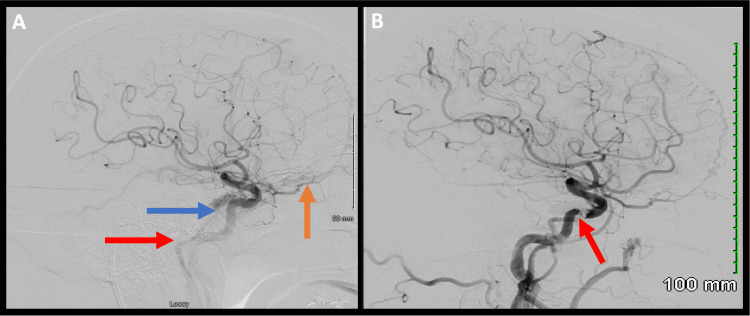
Lateral left ICA angiogram A: Lateral left ICA angiogram showing carotid-cavernous fistula (blue arrow) with early venous drainage to the superior ophthalmic vein (orange arrow) and inferior petrosal sinus (red arrow). B: Lateral left ICA angiogram after coil embolization showing obliteration of the cavernous fistula (red arrow).

A 71-year-old male presented to the clinic with a three-week history of decreased visual acuity in the left eye. Physical examination was significant for left-sided vision loss, lid lag, and chemosis without proptosis. Cerebral angiography revealed a CCF supplied by both indirect internal and external carotid feeding arteries, with drainage into the superior ophthalmic vein and ipsilateral inferior petrosal sinus (Figure 2A). The patient underwent transvenous coil embolization of his left carotid-cavernous fistula using combined TRA and UETV (Figure 2B). The procedure went without complications, and no access site complications were encountered. The patient was discharged on postoperative day 1 with improvement in his visual acuity.

Procedure description

Transradial Access

The radial artery was cannulated using a single-wall technique with ultrasound guidance. A 6 French Merit Prelude Ideal radial vascular sheath (Merit Medical, South Jordan, Utah) was placed approximately halfway into the artery. A radial artery angiogram was performed, and a standard radial cocktail was administered. Under roadmap guidance, the dilator and wire were reinserted to allow the 23-cm sheath to be inserted fully, placing the sheath tip in the brachial artery. IV heparin was administered. A Simmons 2 Catheter was used to selectively catheterize the carotid arteries of interest for cerebral angiography. At the end of the procedure, patent hemostasis of the radial artery was obtained in a standard fashion using a transradial band.

Transvenous Access and Venogram With Coil Embolization of the Cavernous Fistula

A sterile 18-gauge IV was placed in the preoperative area in the cephalic vein of the forearm (Case 1) (Figure 1A) or into the cubital vein of the antecubital fossa (Case 2). The IV was prepped into the sterile field and exchanged for a 16-cm 6 French Merit Prelude Ideal radial sheath using a microwire in the mid-right forearm, following which a venogram was performed. Under roadmap guidance, a 6 French guide catheter, navigated over a 5 French diagnostic catheter and hydrophilic guidewire, was navigated from the subclavian vein into the internal jugular vein on the side of the fistula. The guide catheter was navigated to the origin of the inferior petrosal sinus, and the remainder of the case proceeded in a standard fashion. Following the procedure, the sheath was removed, and hemostasis was obtained using gauze and a compressive bandage.

## Discussion

The authors present the cases of two patients undergoing combined TRA and UETV for CCF embolization. UETV has been well-explored in interventional cardiology literature, but there are few studies in the neurointerventional literature, as UETV is still an emerging strategy in the field [1]. Combined arterial and venous access in the arm has gained additional traction in interventional cardiology due to the possibility of patients continuing anticoagulation and thereby avoiding thromboembolic events that might occur when anticoagulation is held, which is particularly relevant in cardiac patients. However, the question emerged as to whether or not there was a benefit with upper extremity access in a purely transvenous procedure [5]. One study compared the safety of forearm TVA with common femoral, jugular, and subclavian venous access. This group found shorter fluoroscopy times, similar radiation doses, and no complications in the arm group, with 3% of patients experiencing minor complications in the common femoral, jugular, and subclavian venous access groups, including hematoma and carotid puncture [6]. There was a 9% failure rate using upper extremity TVA due to small venous size, occlusion, or inability to access [7]. Another prospective study on 1007 patients analyzed arm and femoral access for cardiac procedures and found shorter procedure duration and fluoroscopy time, with lower radiation doses and rates of hematoma in the arm group [1].

No access site or procedural complications were noted in either of the authors’ cases. Obtaining venous access with an IV in the preoperative area likely decreased the time required to obtain venous access compared to standard TVA. Both patients were able to benefit from early mobility and ambulation compared to standard transfemoral approaches. Combined TRA and UETV is a feasible, promising access strategy for patients and may confer the same safety and patient satisfaction outcomes that have been seen with TRA in neurointerventional procedures [4,8]. Further studies are needed to elucidate the exact impact this strategy has on patient outcomes and satisfaction.

## Conclusions

Upper extremity transvenous access (UETV) is a promising access site that may confer the same safety, cost, and preference benefits as its arterial counterpart, transradial access (TRA). Both presented patients were able to benefit from early mobility and ambulation compared to standard transfemoral venous approaches.
